# 3D printed guide-assisted percutaneous screw fixation for minimally displaced scaphoid waist fractures with delayed diagnosis or presentation

**DOI:** 10.1186/s12891-024-07243-1

**Published:** 2024-02-10

**Authors:** Cunmin Rong, Qinglin Zhang, Shaobo Zhu, Guanghui Zhang, Junhao Zeng, Qingluan Han, Yang Guo

**Affiliations:** 1 Department of Hand and Foot Surgery, The Affiliated Hospital of Jining Medical University, Jining, 272029 Shandong China; 2 Department of Hand Surgery, Beijing Jishuitan Hospital, Beijing, 100035 China

**Keywords:** Scaphoid fracture, Percutaneous, 3D printed, Delayed diagnosis, Delayed presentation

## Abstract

**Objectives:**

To Investigate the value of 3D printed guide-assisted percutaneous management of minimally displaced scaphoid waist fractures(Herbert’s B2) with delayed diagnosis or presentation.

**Methods:**

From October 2018 to February 2022, 10 patients with established delayed diagnoses and presentation of minimally displaced scaphoid waist fractures were treated with 3D printed guides assisted with percutaneous internal fixation without bone grafting. This technique was based on the patient’s preoperative CT and imported into the software. Based on Boolean subtraction, the most centralized screw placement position was identified and a customized guide was produced. Intraoperative percutaneous insertion of the guide wire was assisted by the custom guide.

**Results:**

All 10 patients were successful in one attempt. The fractures healed at a mean of 7.7 weeks postoperatively (range 6–10 weeks). At a mean follow-up of 7.7 months (6–13 months), patients had excellent recovery of wrist function with minimal pain reduction. There were no major postoperative complications and the patients all returned to their previous activities before the injury.

**Conclusions:**

Percutaneous internal fixation based on 3D printed guides is a safe and effective technique for delayed diagnosis or presentation of patients with minimally displaced fractures of the scaphoid waist. This method allows for easy insertion of screws and avoids multiple attempts.

## Introduction

Delayed diagnosis of scaphoid fracture frequently occurred, due to the unique and complex anatomical structure of the scaphoid [1]. Untreated acute scaphoid fracture can lead to nonunion and wrist arthritis for several years [2]. However, lack of medical and radiological diagnosis and delayed presentation are also important factors contributing to the nonunion of scaphoid fractures. When the diagnosis and presentation were delayed by 4 weeks, the rate of bone nonunion was as high as 40%, whereas when it occurred within 4 weeks, the rate of fracture nonunion was only 3% [3]. Therefore, these neglected fractures require prompt and careful treatment to prevent chronic sequelae [4]. 

There is no consensus on the optimal management of scaphoid fractures with delayed diagnosis or presentation [5, 6]. Some reports suggest that management of these fractures should be similar to non-healing fractures [2, 4]. However, open reduction and internal fixation with bone grafting is considered the standard treatment of scaphoid delayed unions [7–9]. Recently, percutaneous screw fixation without bone grafting has been proposed for stable and minimally displaced cases [10, 11]. 

However, the freehand percutaneous insertion of the screw to the central part of the scaphoid still requires a high level of skill. The recent development of 3D printing technology has enabled percutaneous screw insertion for the treatment of acute scaphoid waist fractures, and have obtained better clinical results [12–16]. This study aimed to evaluate the clinical and imaging outcomes of 3D printed guides assisted percutaneous treatment of delayed diagnosis or presentation of minimally displaced scaphoid waist fractures.

## Patients and methods

### General data

We retrospectively reviewed 10 consecutive patients with delayed diagnosed or unions of the scaphoid fractures (Herbert’s B2) treated between October 2018 to February 2022, treated by percutaneous screw fixation without bone grafting assisted by 3D printed guides.

There were 7 males and 3 females, with a mean age of 31.4 years (25 ~ 53 years). The mean time from injury to surgery was 53.3 days (23–150 days). There were 8 cases of right scaphoid fracture and 2 cases of the left; 7 of them were smokers and 8 cases of the dominant hand. One patient had a combined distal radius fracture, and the distal radius fracture was treated with freehand percutaneous internal fixation.

Of the 10 patients, 5 patients did not go to the hospital originally, and went to the hospital because of the persisting pain. 3 patients, during the initial investigation in the clinic, either could not obtain radiographs in the acute phase or did not take the radiographs. The remaining 2 patients were informed of fractures during radiological examinations performed in the acute period but did not follow medical advice to immobilize the wrists. All the above patients underwent CT examinations parallel to the longitudinal axis of the scaphoid in our out patient clinic to confirm the delayed union of fracture [17]. 

Inclusion criteria of our study were Herbert’s B2 type, minimally displaced fracture and cystic or sclerotic change < 5 mm. As further reduction or (and) internal fixation with bone grafts may be required, exclusion criteria were dorsal intercalated segmental instability (DISI) deformity, avascular necrosis of the proximal pole, and humpback deformity. History of previous trauma or previous surgical procedures on the affected wrist was also excluded.

### Surgical technique

**Fig. 1:**
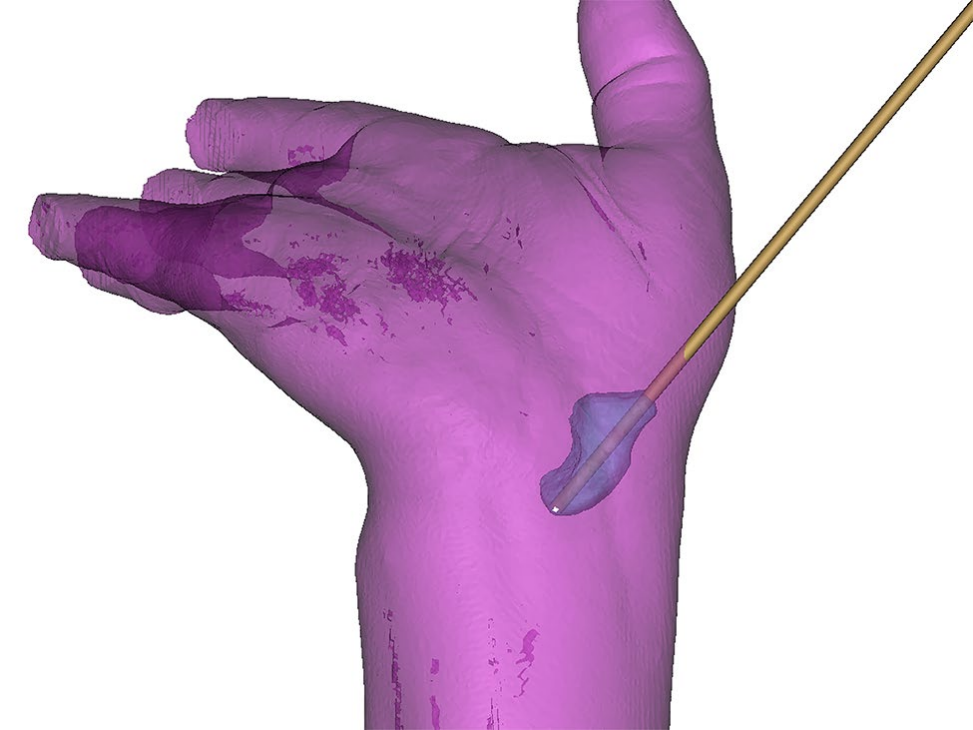
The optimal screw position was obtained for the most central region of the scaphoid

All patients obtained preoperative CT scans of the wrist in supination, hyper-extension, and ulnar deviation positions (GE, USA, scan slice thickness 0.625 mm, continuous scans without gaps) from the mid-forearm to the proximal interphalangeal joint of the fingers. The saved data in DICOM format were processed (E-3D software 17.0, Central South University, China) to obtain the screw position in the most central portion of the scaphoid according to the Boolean subtraction (Fig. 1) [12, 13]. After reconstruction, the palmar and dorsal parts of the guide that can be tightly combined together were obtained, and the guide data were saved as .stl files. To guide the intraoperative screw selection, the optimal screw length could be measured. 3D printer (SLA600, Suzhou Polly Company, China) was used to print the guide plate, using biocompatible material (PollyPolymer, Suzhou Polly Company, China).

**Fig. 2:**
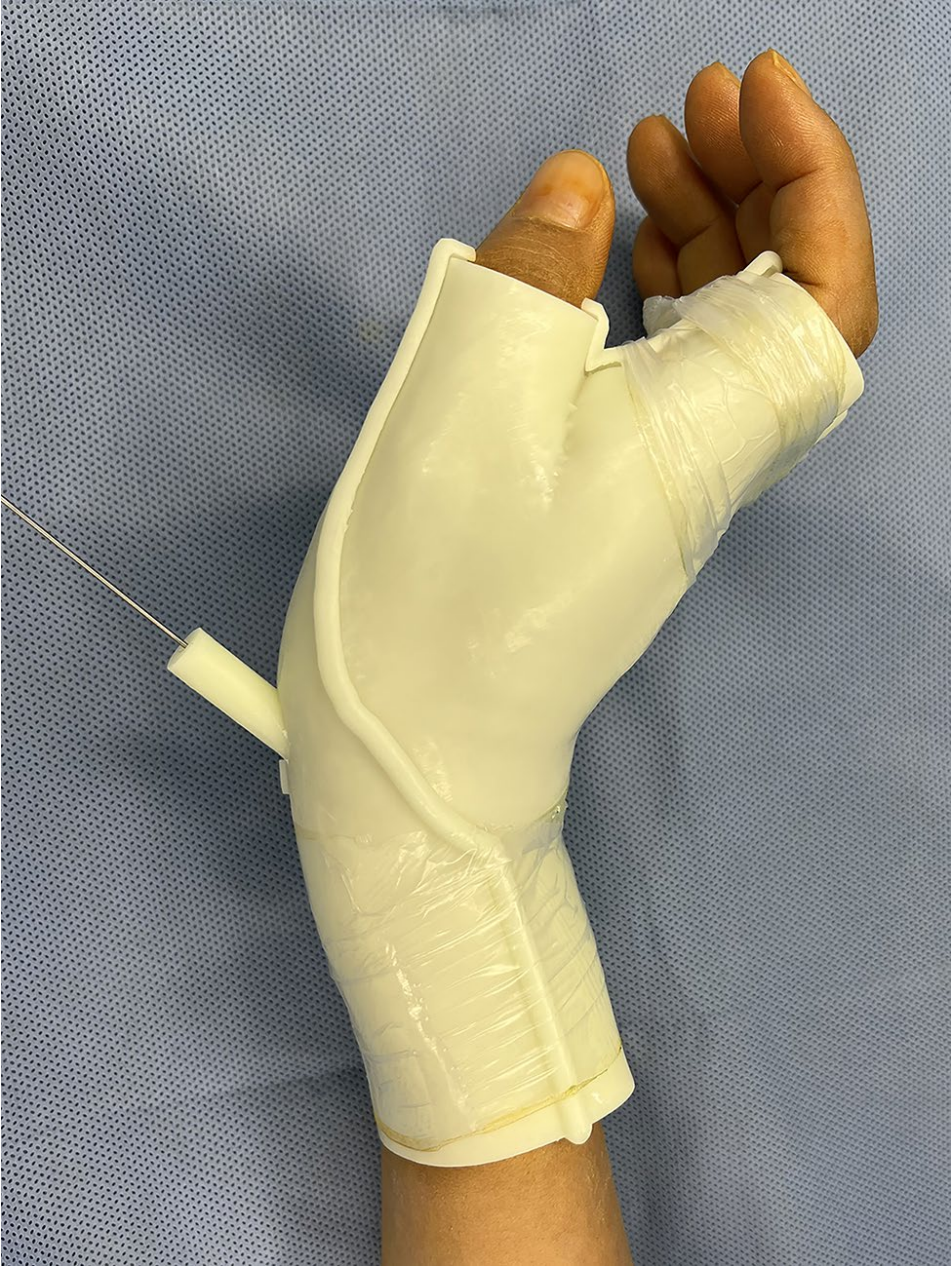
3D printed guide assists in guiding the insertion of the needle from the scaphoid tuberosity

**Fig. 3:**
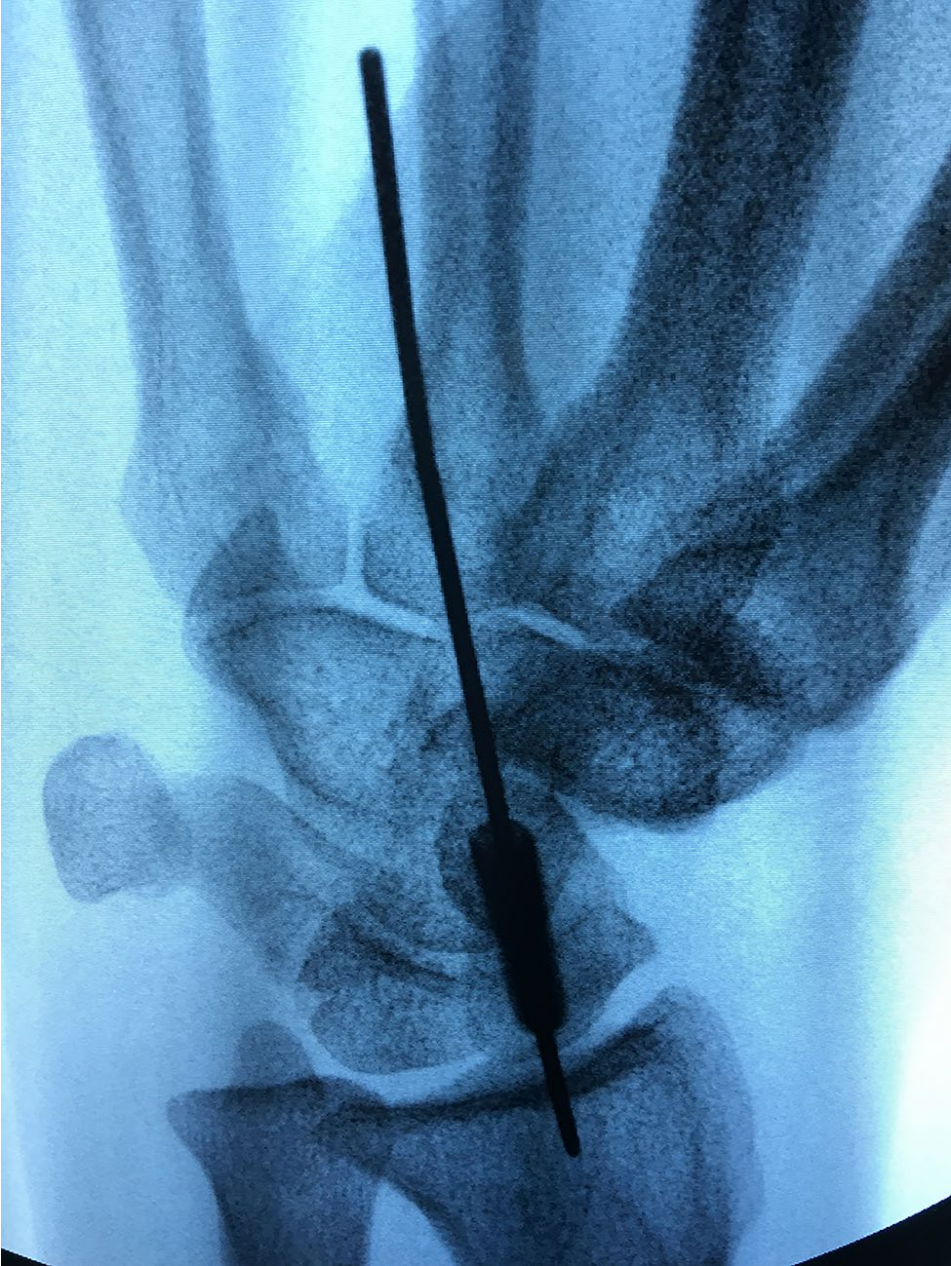
Intraoperative fluoroscopy showing the optimal position and length of the screw in the semi-supinated oblique position of the wrist joint

The guide was sterilized with hydrogen peroxide solution at a low temperature before operation. The surgery was performed under brachial plexus anesthesia and a tourniquet was not necessary. Intraoperatively a guide pin (1.0 mm diameter) was inserted through the scaphoid tubercle under the guidance of the 3D-printed guide (Fig. 2). Fluoroscopy (GE Elite MiniView, GE, USA) confirmed the optimal guide pin position (anteroposterior (AP), lateral and semi-supinated oblique images). A 0.5 cm incision was made at the scaphoid tubercle. A headless compression screw of appropriate length was inserted (3.5 mm diameter, Wuxi Baide, China). Fluoroscopy confirmed no penetration was noted at both the proximal and distal pole of the scaphoid(Fig. 3). The incision was closed with steri-strips before bandaging. The wrist was immobilized with a thermoplastic short arm splint for 2 weeks, allowing gentle movement of the fingers postoperatively.

### Outcome evaluation

The scaphoid is considered healed if the trabecular bridging exceeds 50% of the fracture site [18]. Plain radiographs were obtained at week 4 postoperatively and then every 3–6 weeks until fracture healing was determined. Final confirmation of fracture healing were confirmed by CT scan. Then daily activities can be gradually resumed.

**Fig. 4:**
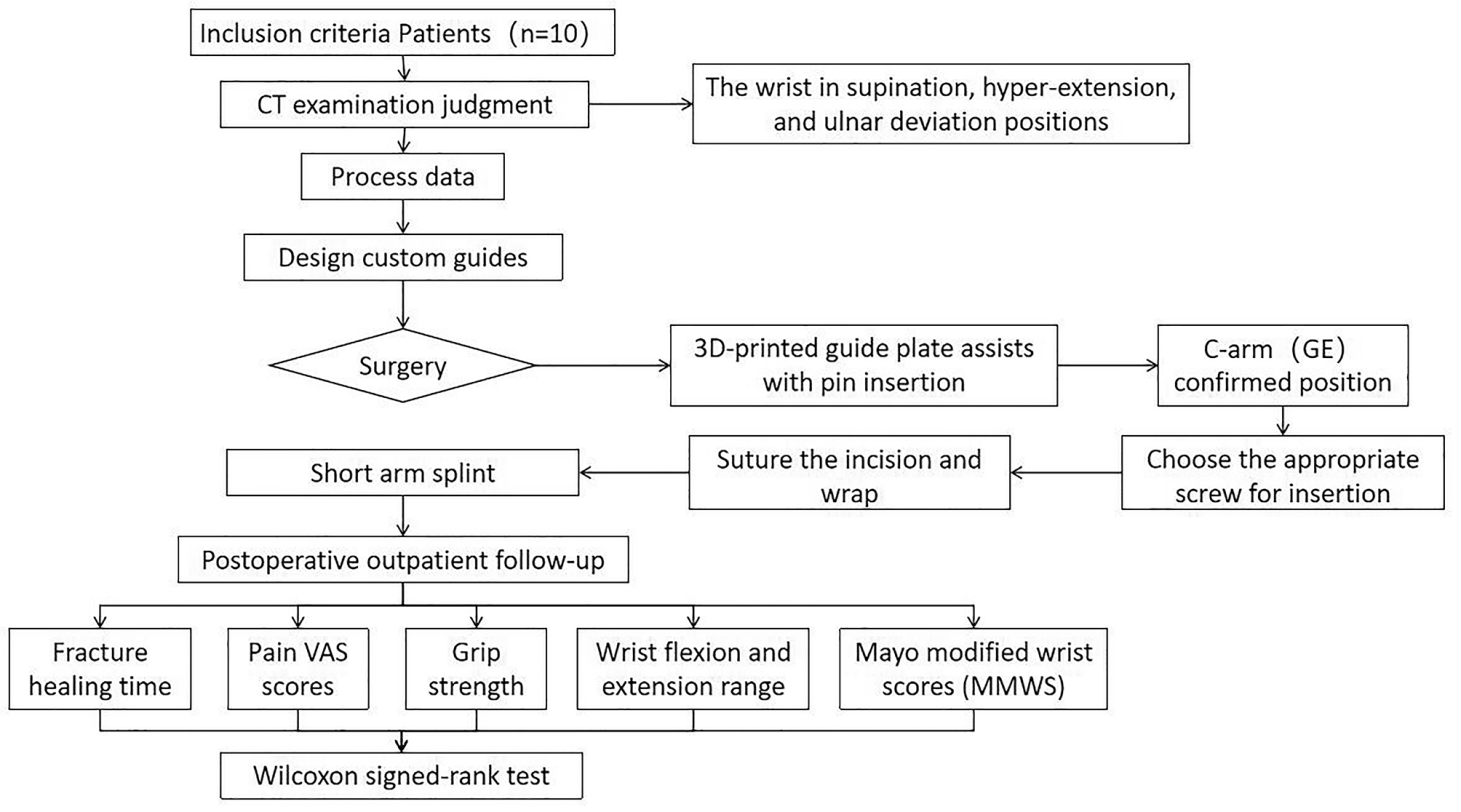
Flow chart

Fracture healing time was recorded in all patients. Clinical outcomes were assessed for all patients, including pain VAS scores, wrist flexion and extension range of motion, grip strength, and Mayo modified wrist scores (MMWS), and data were statistically analyzed using Wilcoxon signed-rank test to compare clinical values at preoperative and final follow-up. (Fig. 4)

## Results

Ten patients with delayed diagnosis or presentation of scaphoid waist fractures were included in this study, and all of them were performed with percutaneous internal fixation assisted by 3D printed guides without bone grafting. The minimum follow-up time for patients was 6 months, with a mean of 7.7 months (range 6–13 months). All patients did not have any postoperative complications such as infection or nerve injury.

**Fig. 5:**
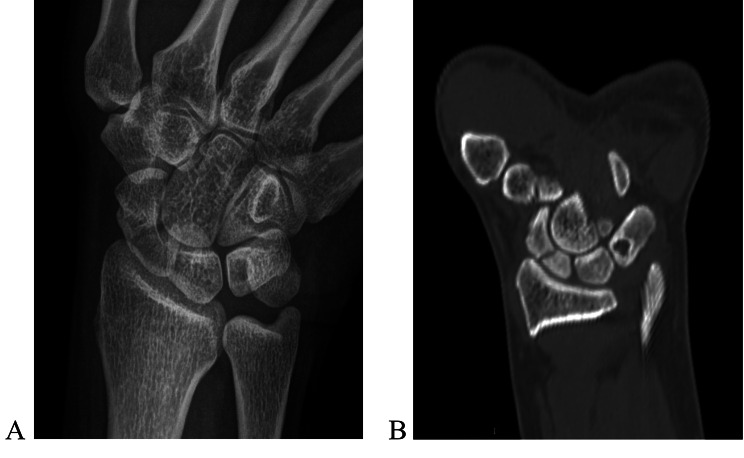
Preoperative radiograph (**A**) and coronal CT of the right wrist (**B**), showing a 35-year-old woman with a delayed-presentation scaphoid waist fracture 72 days after injury

**Fig. 6:**
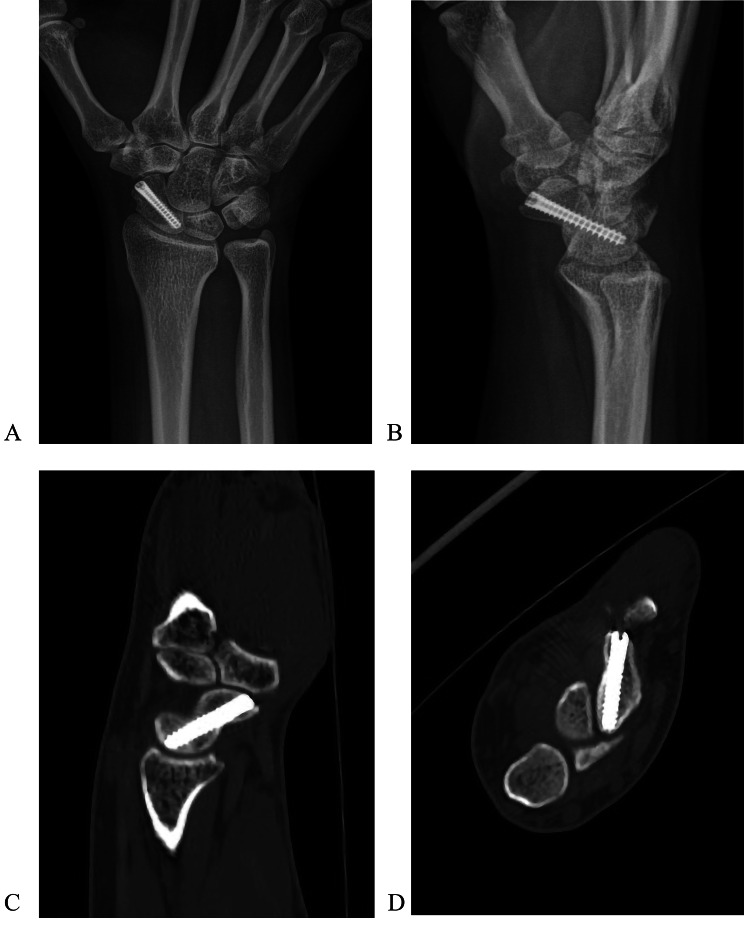
Anteroposterior (**A**), lateral (**B**) radiographs, sagittal (**C**), and horizontal (**D**) CT at 2 months postoperatively showed that the fracture was healed, and the screw was positioned in the most central region of the scaphoid

All guide-wires in the 10 patients were inserted in the desired place in the scaphoid with one attempt. The mean overall operative time was 30.7 min(range 21–48 min). Radiologic evaluation showed that 5 (50%) scaphoid healed in 6 weeks postoperatively. 7 (70%) healed in 8 weeks postoperatively, 8 healed in 10 weeks postoperatively, and 100% of the scaphoid bones healed in 12 weeks postoperatively (Fig. 5), including 7 smokers. CT confirmed fracture union and also demonstrated that the screws were centrally located and optimally positioned, with no screw penetration(Fig. 6). There was no nonunion of the fracture.

**Fig. 7:**
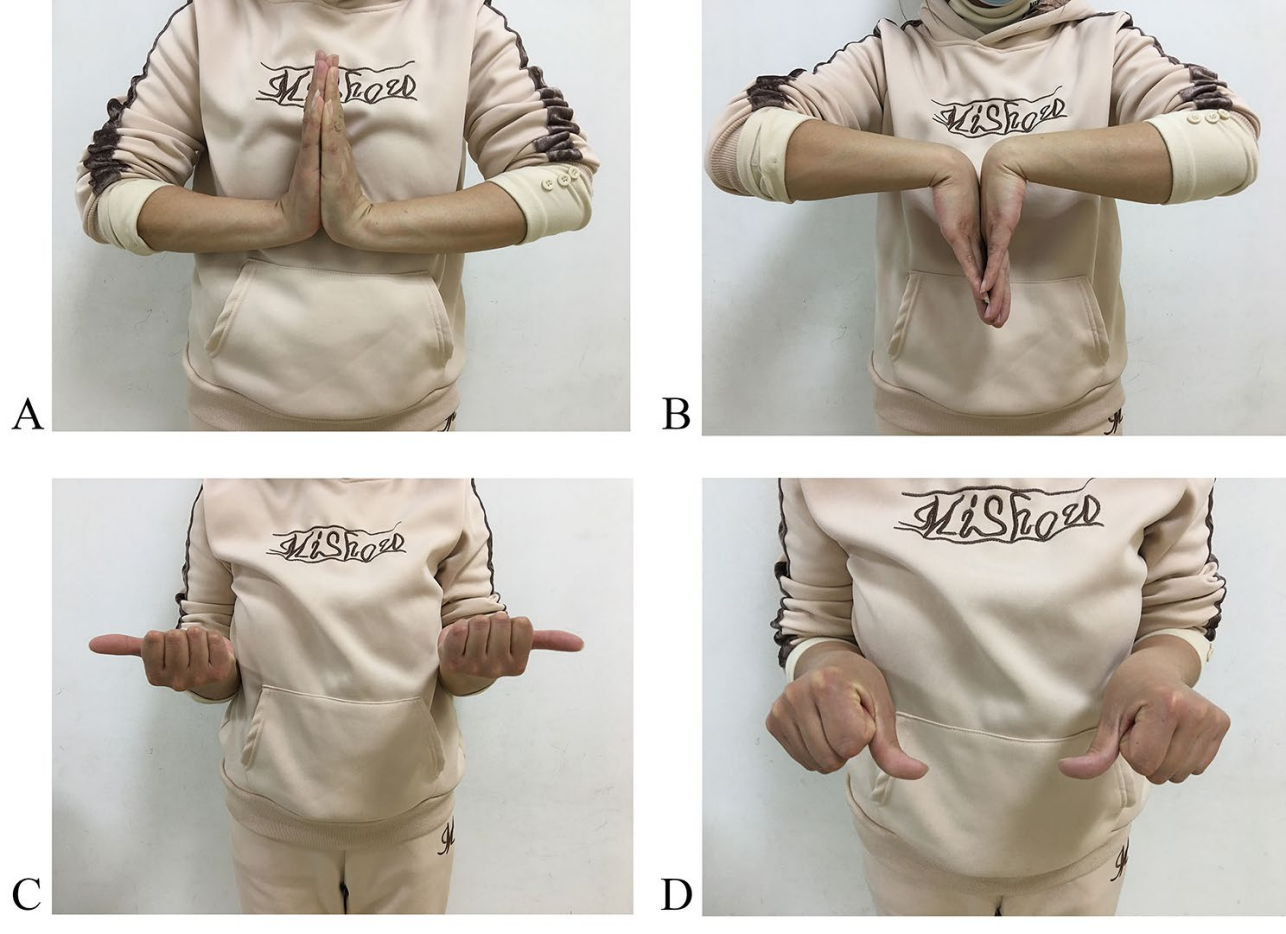
At the 10-month postoperative follow-up, the right wrist joint was 90°of extension (**A**), 85°of flexion (**B**), 90°of supination (**C**), and 90°of pronation (**D**)

Eight hands were dominant. The preoperative and final follow-up measurements are listed in Table 1. Generally, the patient’s wrist function recovered positively. Grip strength was assessed as a percentage of contralateral strength (Jamar dynamometer, SI Instruments, Hilton, SA 5033, Australia). The mean postoperative grip strength improved by approximately 40% from preoperative compared to the contralateral wrist, and wrist flexion and extension range of motion improved by 30–40% (Fig. 7). The pain was minimized.

**Table 1:** Preoperative measurements and final follow-up outcome measurements

Assessment	Preoperative	Postoperative	Z-score	*P*-value
Pain VAS^∗^ score	5.50(4.00,6.00)	0.00(0.00,1.00)	-2.820	<0.01
Wrist extension(°)	46.00(41.25,54.00)	88.00(85.00,90.00)	-2.803	<0.01
Wrist flexion(°)	52.00(43.75,56.25)	90.00(86.50,90.00)	-2.812	<0.01
Grip strength (% of contralateral)	54.55(51.05,67.28)	97.90(85.03,108.00)	-2.803	<0.01
Modified Mayo wrist score	55.00(50.00,60.00)	100.00(95.00,100.00)	-2.844	<0.01

∗ VAS: visual analogue scale

Wilcoxon signed-rank test

Wrist function was assessed using the Modified Mayo Wrist Score (MMWS). The mean postoperative MMWS was about 40% more improved than the preoperative assessment. All 10 patients returned to their previous work.

## Discussion

Our management achieved healing in 10 patients with minimally displaced scaphoid waist fractures with delayed diagnosis or presentation, who had been injured for less than 5 months and had no bone graft. And the successful insertion of the guide pin in the central region of the scaphoid was achieved in one attempt.

A study of 52 patients with neglected scaphoid fractures showed that 37 cases (71%) were neglected because the possibility of a scaphoid fracture was not considered, and clinical signs of a scaphoid injury were not evaluated carefully by the doctor [1]. Fractures may not be visible on the “scaphoid series” X-rays immediately after injury [1, 19]. Delayed treatment increases the risk of non-union by up to 40-88% [4, 19]. A clinical and epidemiological study of 363 Chinese patients with non-healing scaphoid fractures by Yin et al. showed that non-healing of waist fractures was 280 cases (76.5%), of which 53% nonunion was mainly due to delayed presentation [20]. Grewal et al. managed 28 patients with scaphoid fractures diagnosed between 6 weeks and 6 months using cast immobilization for 11–14 weeks, with a healing rate of 82% [19]. However, Chen et al. reported that of 30 patients with scaphoid fractures between 2 weeks and 5 months post-injury, 87% underwent open surgery and 97% of these fractures healed [4]. 

Many patients with delayed diagnosis and presentation of scaphoid fractures have fracture patterns similar to nonunion, so the management plan will be similar to fracture nonunion [2, 4]. Open reduction and internal fixation with bone grafting is considered the reference treatment of scaphoid nonunions and delayed unions [7–9]. However, the superior results and high healing rates of percutaneous internal fixation techniques have been reported in several studies [21–23]. Wong et al. treated scaphoid fracture patients for an average of 10 weeks (3–28 weeks) after injury with screw or Kirschner wire percutaneous fixation, and all fractures healed smoothly [24]. A single-center experience reported that internal fixation alone was effective in the treatment of subacute proximal scaphoid fractures (14.2 ± 5.7 weeks post-injury) [25]. . In recent years, percutaneous internal fixation with double screws has been promising in the treatment of acute scaphoid waist fractures, delayed healing, and non-healing, and double screw treatment has a high rate of healing and a low rate of reoperation [26]. However, Single- and double-screw fixation in a cadaveric model of scaphoid nonunion showed similar construct stiffness, failure loads, and loads at 1-mm and 2-mm displacements. Therefore, the theoretical benefits of double-screw fixation should be evaluated against the morphological limitations of placing 2 screws in the scaphoid nonunion [27]. Therefore, the currently generally accepted indications for the fixation of screws without graft are delayed union and humpback deformity, proximal necrosis, DISI, and ununited without significant bony loss.

Single screw fixation of the central long axis is still a common method for the management of scaphoid fractures [28]. The central long-axis screw has good biomechanical stability [4, 24]. That’s why we chose to place a central zone screw. However, the technique of inserting screws in the central region of the scaphoid with free hands is challenging [12–15, 29]. Although guidance-assisted techniques have been applied to guide percutaneous scaphoid screw insertion, it still requires hand-eye coordination of the surgeon [29]. Studies have demonstrated the reliable viability of 3-D printed guides by comparisons of pre-determined screw positions and final screw positions from CT scans of cadaveric specimens [13]. Similar results to the freehand approach have been shown in terms of final screw position, with reduced operative time and fluoroscopy time, and reduced costs compared to the robotic-guided approach [13, 14, 29]. Recent studies have shown that 3D guide-assisted percutaneous internal fixation for acute scaphoid waist fractures can be performed with significantly less operative and fluoroscopic time and less attempts [12–16, 29], although there may be no significant difference in accuracy [14]. In a recent study by Marcano-Fernández et al. of 3D printed custom guides for percutaneous internal fixation in patients with acute scaphoid fractures, only 1 out of 10 patients was not included. The reasons may be the long time from CT scanning to surgery (> 1 week), a significant reduction in soft tissue swelling, and the loosening of the guide during the procedure. This demonstrated the high reliability and reproducibility of the technique [14]. Nevertheless, the fact that our patient was not an acute patient and the swelling of the limb did not change noticeably may also account for the success of one attempt in all our patients.

Instability is a major factor affecting the fracture healing process. well-aligned bone nonunion without disturbed vascularity requires only a rigid fixation, percutaneous reaming provides adequate clearing at the site of bone nonunion, and the stability provided by screws resulted in minimally displaced fracture healing.^7^ The mean healing time was 7.7 weeks (5–12 weeks), which was shorter than the time reported with previous percutaneous fixation treatments. We thought this to the high purchasing power of the screws without the repeated insertion attempts of the guide pins. Wrist motion and grip strength were significantly improved (*P*<0.01). Minimally displaced scaphoid fractures did not require further reduction so the preoperative surgical pathway can be planned accurately and the guide plate can be meaningful for the surgical procedure. However, we are not sure if this is associated with the success of one-attempt screw insertion, and it is possible that our selection of patients, injury-to-operative time, dominant hand, and type of fracture were not the same as other authors.

While there is a cost for the 3D-printed guides (only $50–100), the cost is gradually decreasing, as the technology improves. However, the 3D printed guide plate has the advantages of lower cost, less steep learning curve, higher acceptance by hand surgeons and patients, and easier promotion compared with robot assisted percutaneous fixation of scaphoid fracture [29]. 

The limitation of this study is a retrospective study of clinical cases without a control group. The number of cases is relatively small and requires long-term follow-up studies. Although the increased cost of 3D printed guides, the reduction in procedure time and fluoroscopy time can compensate for the loss. While this was an attempt at a fracture with minimal displacement, with further research, we believe that more complex fractures are potentially going to gain resolution. However, it is worthwhile to perform the management of a large sample of patients with scaphoid fractures who are not fresh and do not have significant DISI deformity or humpback deformity by applying this technique.

## Conclusion

Based on the above information and clinical observations, incisional bone grafting may not be necessary for waist fractures of the scaphoid (23–150 days) with delayed diagnosis and presentation. Percutaneous fixation with the assistance of 3D printed guides is feasible, fracture healing can be obtained, and wrist function is significantly improved. However, the selection of patients is important.

## Data Availability

The detailed data in the article can be obtained from the corresponding author: rongcunmin@sina.com.
